# Gas Chromatography–Mass Spectrometry Based Approach for the Determination of Methionine-Related Sulfur-Containing Compounds in Human Saliva

**DOI:** 10.3390/ijms21239252

**Published:** 2020-12-04

**Authors:** Justyna Piechocka, Monika Wieczorek, Rafał Głowacki

**Affiliations:** Department of Environmental Chemistry, Faculty of Chemistry, University of Lodz, 163 Pomorska Str., 90-236 Łódź, Poland; m.wronska17@onet.eu

**Keywords:** amino acid, aminothiol, cysteine, gas chromatography–mass spectrometry, homocysteine, homocysteine thiolactone, human saliva, methionine, *N*-trimethylsilyl-*N*-methyl trifluoroacetamide, sulfur amino acid

## Abstract

Gas chromatography–mass spectrometry technique (GC-MS) is mainly recognized as a tool of first choice when volatile compounds are determined. Here, we provide the credible evidence that its application in analysis can be extended to non-volatile sulfur-containing compounds, to which methionine (Met), homocysteine (Hcy), homocysteine thiolactone (HTL), and cysteine (Cys) belong. To prove this point, the first method, based on GC-MS, for the identification and quantification of Met-related compounds in human saliva, has been elaborated. The assay involves simultaneous disulfides reduction with tris(2-carboxyethyl)phosphine (TCEP) and acetonitrile (MeCN) deproteinization, followed by preconcentration by drying under vacuum and treatment of the residue with a derivatizing mixture containing anhydrous pyridine, *N*-trimethylsilyl-*N*-methyl trifluoroacetamide (MSTFA), and trimethylchlorosilane (TMCS). The validity of the method was demonstrated based upon US FDA recommendations. The assay linearity was observed over the range of 0.5–20 µmol L^−1^ for Met, Hcy, Cys, and 1–20 µmol L^−1^ for HTL in saliva. The limit of quantification (LOQ) equals 0.1 µmol L^−1^ for Met, Hcy, Cys, while its value for HTL was 0.05 µmol L^−1^. The method was successfully applied to saliva samples donated by apparently healthy volunteers (*n* = 10).

## 1. Introduction

Methionine (Met)-related compounds, such as homocysteine (Hcy), Hcy-thiolactone (HTL), and cysteine (Cys), have received continuing attention due to their physiological importance and considerable implications in so-called civilization diseases. Frequently, disturbed Met-related sulfur-containing compounds metabolism is associated with cardiovascular, cancer, and neurodegenerative diseases, among others [1,2,3,4,5]. Despite all extensive research is being done into the connection between the several human diseases and the above-mentioned sulfur compounds’ metabolism disorders, too little is still known about their physiological and pathological role in living systems. Therefore, it seems to be essential to provide more robust and versatile platforms for a comprehensive assessment of Met and metabolically related compounds metabolome in order to increase knowledge about their role in the human body, in the near future. In addition, it would be desirable to provide new analytical tools facilitating large-scale screening, from the standpoint of an effective fight against high morbidity and mortality from these diseases.

Over the last decades, the gas chromatography–mass spectrometry technique (GC-MS) has been proved to be one of the most powerful analytical tools for biofluids analysis. Most probably, due to high-throughput potential, sensitivity, specificity, and great resolution, along with high degrees of reproducibility and accuracy, the GC-MS receives continuing interest. So far, numerous methods based on GC-MS for HTL, Met, Hcy, and Cys determination have been developed. In relation to gold standards in the field of clinical, toxicological, and forensic science, which include blood (plasma, serum) and urine tests, a few GC-MS assays have been designated for simultaneous assessment of urinary and/or blood plasma Met, Hcy, and Cys [6,7,8,9,10,11,12,13,14,15]. Interestingly, none of them allows the sensitive determination of the above-mentioned aminothiols and Met in human saliva. To the best of our knowledge, only one GC-MS assay, applied for identification and quantification of human salivary and urinary HTL, has been developed so far [16]. Since saliva is currently supposed as an alternative to blood and urine in the diagnosis of some diseases [17,18,19,20,21,22,23], we have decided to take on the challenge of demonstrating the usefulness of GC-MS in saliva analysis for Met-related sulfur-containing compounds. Furthermore, taking into account recent directions in bioanalysis, the present topic appears as very important. It seems to be essential to put efforts into developing new, simple, and low-price analytical tools facilitating large-scale screening of civilization diseases. Moreover, searching for new sources of biomarkers, which can be obtained in a non-invasive and non-intrusive way in order to encourage the general public to get through regular checkups, becomes crucial. Most probably non-invasive and non-intrusive nature of the saliva sampling process as well as the fact that various drugs and metabolites can be detected in saliva with high correlation to plasma/ urine levels have contributed to its growing popularity [17,18,19,20,21,22,23]. According to several authors, the diagnosis of many pathological conditions in the human body could thus benefit from the analysis of saliva specimens. Therefore, our efforts were predominantly attributed to the development of a highly effective analytical tool based on GC-MS for simultaneous determination of salivary Met, HTL, and total Cys and Hcy content as well as the application of the assay to real samples in order to confirm or exclude the performance of the method. In particular, the article discusses essential steps, with some justification, which were taken to achieve the intended goal. Moreover, the advantages, pitfalls, and limitations of the GC-MS assay are mentioned.

## 2. Results and Discussion

It is commonly known that biological fluids, to which saliva belongs, consist of a large number of components representing widely varied structures and physicochemical properties. Despite technological advances in analytical and bioanalytical techniques, the complexity of biofluids attributes to tremendous challenges to analysts since a successful analysis of a crude sample still cannot be achieved without appropriate sample pretreatment. In the case of assays based on separation techniques, proper sample handling and management combined with separation and detection conditions play a key role in the quality of obtained results. Therefore, a few experiments were performed in order to provide the GC-MS assay reliability. Considerable attention has been put on optimizing sample preparation and chromatographic conditions during new method development. In general, commonly known approaches, of which the usefulness and validity have already been established, were utilized to achieve the intended purpose. In addition, an important aspect of research work was to stick to principles of green chemistry and to keep in mind that “Everything should be made as simple as possible, but no simpler”.

### 2.1. Sample Preparation

In the presented study, the GC-MS method has been designed to determine salivary Met, HTL, and total thiols content, including Hcy and Cys. Sample preparation involves simultaneous disulfides reduction with tris(2-carboxyethyl)phosphine (TCEP) and saliva deproteinization by addition of acetonitrile (MeCN). The next step is preconcentration by drying under vacuum and chemical modification by treatment of the residue with a mixture containing anhydrous pyridine, N-trimethylsilyl-N-methyl trifluoroacetamide (MSTFA), and trimethylchlorosilane (TMCS). The choice of essential sample preparation steps was made upon knowing the chemical properties of the analyte(s) as well as the requirements and capabilities of the implemented analytical technique. In general, these steps are typical of such kind of methods. Different analytical tools have been employed to determine optimal conditions for sample preparation. In the presented study, such an approach has been notably helpful in producing significant results in a rapid and efficient way, minimizing chemicals and plastic consumption, reducing labor efforts, etc. The method is based on high-performance liquid chromatography (HPLC) with pre-column derivatization with 2-chloro-1-methyllepidinium tetrafluoroborate (CMLT) and spectrophotometric detection (UV) was used during the experiments concerning optimization of disulfide bonds reduction [24]. Experiments concerning optimization of analyte preconcentration were conducted with the use of both CMLT-based methodology and the method involving liquid–liquid extraction, lyophilization, and derivatization with MSTFA followed by GC separation [16]. Our earlier studies have indicated that these assays provide a useful tool for quantification of Hcy, Cys, and HTL in human saliva, respectively. At the stage of searching the most suitable deproteinization conditions, an approach utilizing UV spectroscopy was employed [25]. It is widely known that proteins naturally adsorb light at 280 nm, allowing direct measurements of their concentration in a quick and highly reproducible way. Indeed, spectrophotometry enables their convenient monitoring, provided that relatively high concentrations are studied. In the present study, a standard solution of human serum albumin (HSA) at 5 g L^−1^ concentration, instead of saliva, was used. Importantly, such a neat solution was provided since the chemical composition of the proteins and their structures, as well as a number of other saliva components that also absorb light at a specified wavelength, could artificially increase the result calculated from the absorption readings. The HSA solution was treated with appropriate protein precipitating agents. Subsequently, the precipitates were removed by centrifugation and the purity of obtained solution was analyzed assuming that any protein contamination gives rise to an increase in absorption at 280 nm. In fact, both the clarity of obtained solutions and the measurements of the absorption of the water-MeCN supernatants at 280 nm indicated the effectiveness of the protein precipitation. Then, experiments were performed using the described herein procedures based on GC-MS measurements (see Section 3.5 and Section 3.6).

#### 2.1.1. Disulfides Reduction

Low molecular mass thiols, to which Cys and Hcy belong, occur in multiple forms in living creatures thank to extreme ability of sulfhydryl group (-SH) to oxidation. According to literature data [26,27,28], the sample should be treated with a reducing agent, which cleaves disulfide bonds and releases all thiols in reduced form in order to assess the concentration of all redox forms in the assay including their total content. Moreover, this step should be carried out before or simultaneously with sample deproteinization to effectively reduce all oxidized thiols and decouple them from proteins in order to avoid loss in the concentration of analytes. In particular, the latter procedure is recommended since non-covalently bonded individuals can be easily released from denatured proteins as well as the risk of analytes binding by large molecules is reduced under denaturing conditions. Taking into account the above-mentioned considerations, in the presented study, saliva samples were simultaneously subjected to disulfide bond reduction and protein removal. Importantly, such an approach was also advantageous from the standpoint of method simplification as it made it possible to reduce essential sample preparation steps.

In the beginning, the type of reductant was selected. Several reducing agents, including dithiothreitol (DTT), 2-mercaptoethanol (2-ME), TCEP, and tris(hydroxymethyl)phosphine (THP), were tested. Among them, thiol moiety-containing reagents, to which DTT and 2-ME belong, were excluded as their application resulted in complex chromatograms hindering analytes separation and plausible identification. In relation to phosphines, TCEP was found to be superior with regard to disulfides reduction efficiency since signal intensities of 2-S-lepidinium derivatives of Hcy and Cys were about five times greater than those registered when THP was used. As a result, TCEP was chosen, as also recommended in the literature [28].

According to the literature data [29], TCEP is generally well soluble in an aqueous medium at nearly any pH, while its water solutions exhibit the greatest stability. When TCEP is dissolved directly in water, the resulting pH is in the range of 2.0–3.0. Therefore, in the presented study, a stock solution of TCEP was prepared in deionized water. The excellent stability of reducing agent at room and elevated temperature has also been shown to be analytically advantageous (see Section 2.1).

**Figure 1:**
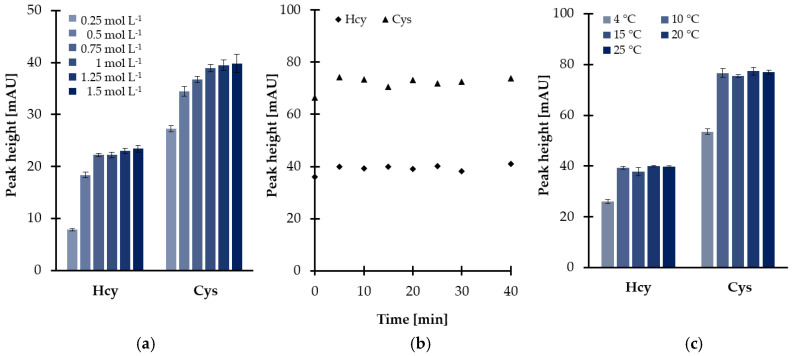
Disulfides reduction yield as a function of (**a**) reagent excess, (**b**) time, and (**c**) temperature, expressed as a peak height of 2-S-lepidinium derivatives of Cys and Hcy. Samples were analyzed according to the procedure based on HPLC-UV measurements [24]. Error bars refer to standard deviation (SD) of the data (*n* = 3).

Further experiments were conducted in order to optimize reduction conditions. The main factor affecting the disulfides reduction yield, namely the quantity of reducing agent regarding thiol, has been optimized initially. Different TCEP solutions at six concentration levels, varied from 0.25 to 1.50 mol L^−1^, were prepared providing the concentration of TCEP in saliva samples in the range of 9.6–57.7 mmol L^−1^. As shown in Figure 1a, the progressive increase of the peak height was observed in parallel with the concentration of TCEP in saliva samples rise from 9.6 to 38.5 mmol L^−1^, then there were no significant changes. Finally, a 1 mol L^−1^ solution of reducing agent was chosen since it has been recognized that a larger excess of TCEP was the source of additional baseline noises. Under these conditions, the reactions were completed in 5 min at room temperature, almost immediately after mixing of the reagents (Figure 1b).

Nevertheless, additional experimental work was undertaken to further optimize the temperature of the reduction reactions as the disulfides reduction step was carried out simultaneously with sample deproteinization. As reported elsewhere [28], protein removal including the addition of polar organic solvents (see Section 2.1.2) followed by centrifugation under reduced temperature helps to obtain a supernatant. In the presented study, it was found that temperature, tested in the range of 10–25 °C, did not markedly affect the effectiveness of the reduction process. As shown in Figure 1c, signal intensities of 2-S-lepidinium derivatives of Hcy and Cys were generally stable throughout the investigated range. Therefore, the centrifuge was kept at the temperature of 10 °C, providing the completion of the process within 5 min and saliva supernatant.

In summary, for routine analysis, 50 µL of saliva was treated with 10 µL 1 mol L^−1^ TCEP in deionized water, representing the final concentration in saliva samples of 38.5 mmol L^−1^. As described above, processing samples for 5 min at 10 °C was sufficient to effectively reduce oxidized thiols with a high level of precision.

#### 2.1.2. Proteins Removal

Human saliva primarily consists of about 99.5% water [17,18,19,20,21,22,28], while the remaining part is made up of sample components that may attenuate the performance of the method. Among them, the presence of proteins in the saliva sample could cause practical problems since the GC-MS system is incapable of accommodating such kinds of biomolecules. Despite saliva is usually considered a low protein abundance matrix, sample deproteinization is indeed needed in order to protect the analytical system against destruction. According to literature data [28], the most commonly used techniques for effective elimination of proteins from saliva involve the addition of inorganic acid or water-miscible organic solvent and ultrafiltration over a cut-off membrane. Based on our previous findings [16,30], some of these methods have not been considered during new method development. In particular, sample acidification was not taken into account since the presence of popular proteins precipitating agents, e.g., perchloric acid (PCA) and trichloroacetic acid, adversely affected the reactivity of the target derivatization agent toward analytes. In addition, the approach utilizing centrifugal concentrators was excluded to minimize plastic consumption and reduce the quantity of the samples.

**Figure 2:**
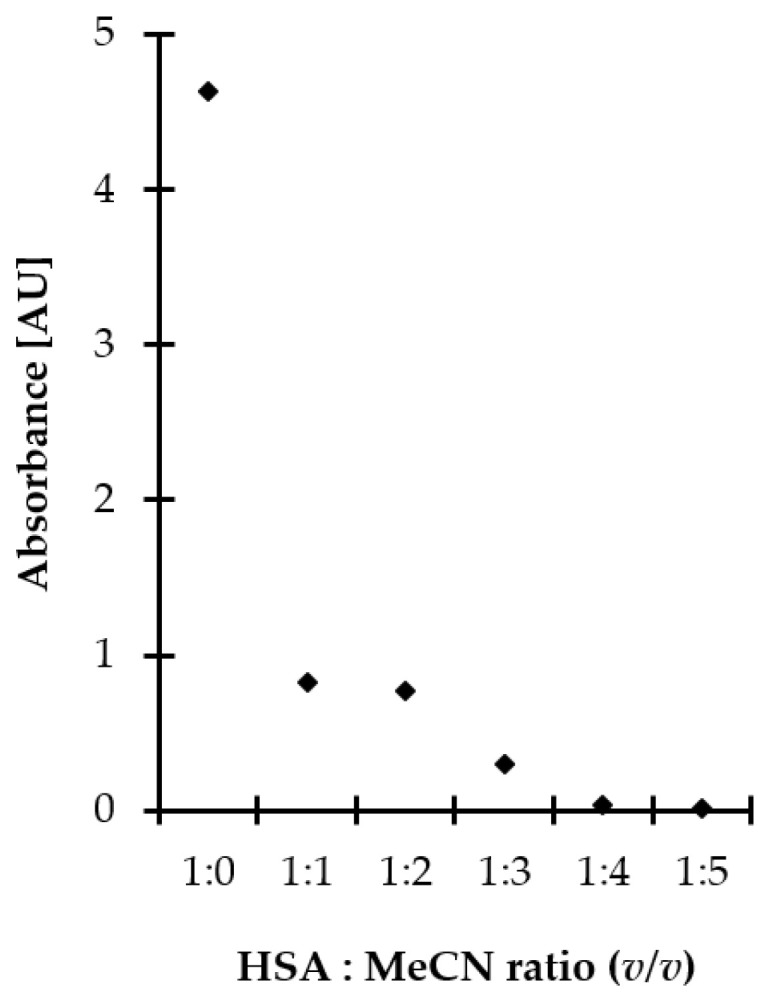
Influence of MeCN amount on deproteinization efficiency, expressed as signal intensity of HSA. Samples were analyzed using the spectrophotometric method based on measurements of the absorption of water–MeCN supernatants of samples at 280 nm.

In the presented study, proteins were efficiently removed from the matrix by applying polar organic solvent to saliva samples, followed by centrifugation under reduced temperature. Moreover, the deproteinization step was conducted concomitant with disulfide bonds reduction contributing to shortening sample preparation time. This approach was also advantageous from the standpoint of increasing recovery of the analytes as non-covalently bonded Met-related sulfur-containing compounds were easier released from denatured proteins, as reported elsewhere [28]. MeCN, typically recognized as the most effective protein precipitating agent among polar organic solvents, was selected. Then, experiments were performed to establish the optimal amount of MeCN. Five different volumes, namely 50, 100, 150, 200, and 250 µL, were tested that amounted to 1–5 times the volume of saliva specimen. As shown in Figure 2, efficient protein removal was achieved by mixing the standard solution of HSA with MeCN, while the minimal crashing ratio of 4:1 was necessary to provide protein-free supernatant. Since 5 g L^−1^ HSA water solution instead of saliva specimen was examined, significantly exceeding the expected amount of salivary proteins [18,28], it was assumed that the 1:4 ratio of saliva to MeCN is optimal. It was also found that the duration of the process, tested in the range of 0–30 min, as well as shaking, did not markedly affect operational efficiency. Since the obtained results were beneficial from the standpoint of workflow simplification, no additional work was undertaken to further optimize the deproteinization step. 

Finally, for routine analysis, 50 µL of saliva was mixed with 200 µL of MeCN, vigorously shaken, and then placed in the centrifuge at 12,000× *g* for 5 min at 10 °C. Under these conditions, a protein-free supernatant was obtained. Notably, analyte dilution was not a limitation as samples were dried under a high vacuum in the subsequent processing step (see Section 2.1.3).

#### 2.1.3. Analytes Preconcentration

In order to counteract sample dilution, caused by adding an excess of the precipitating agent, samples were evaporated to dryness before the subsequent chemical modification step. More importantly, thorough drying of the sample was essential since both derivatization agent and trimethylsilyl derivatives (TMS) are sensitive to hydrolysis [31,32]. In the presented study, saliva supernatants obtained after simultaneous disulfides reduction and saliva deproteinization were evaporated under vacuum at elevated temperature. In order to establish optimal conditions, additional experiments were performed by setting the temperature to various values. As water-MeCN supernatants were dried, temperatures close to the boiling point of MeCN and water were tested specifically. The progressive reduction of time from 45 to 25 min, needed for solvents removal from the samples, was concomitant with temperature rise from 80 to 100 °C. From a purely practical point of view, the explosion and subsequent loss of the sample was not observed under any above-defined conditions. Therefore, the centrifugal vacuum concentrator was kept at the highest possible temperature of 100 °C.

**Figure 3:**
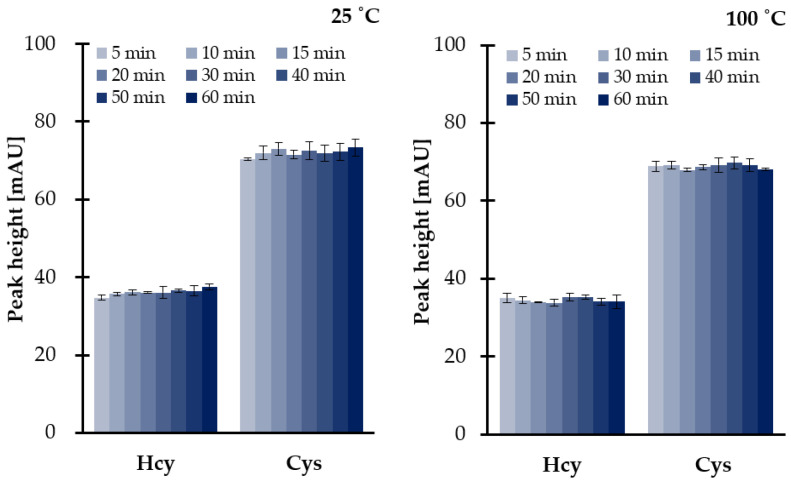
Influence of time and temperature on 1 mol L^−1^ TCEP stability and disulfides reduction efficiency at 25 °C and 100 °C, expressed as a peak height of 2-S-lepidinium derivatives of Cys and Hcy. Samples were assayed according to the previously published method based on HPLC-UV [24]. Error bars refer to SD of the data (*n* = 3).

Since sample decomposition and TCEP could occur, further studies were conducted in order to examine both efficiency of disulfides reduction as well as the stability of analytes under set conditions. Since the reference samples were needed, the same experiments were simultaneously performed at room temperature and at elevated temperature (100 °C). As a result, we were also able to conclude on the stability of the reducing agent (1 mol L^−1^ TCEP). It has been shown that TCEP is not sensitive to temperature (Figure 3), while reduced thiols and HTL remain stable under experimental conditions. We have also found that no more than 6.4% of the reduced aminothiols and other analytes were lost if compared with reference values. The most probable reason for a great stability of reduced thiols at a temperature of 100 °C was attributed to the use of a large excess of 1 mol L^−1^ TCEP preventing their oxidation. Moreover, it is worth mentioning that the presence of an excess of reducing and precipitating reagents has resulted in pH level of saliva changes from neutral to more acidic, providing pH values of 6.5–7.0 and 2.0–3.0, respectively, regardless of saliva donors. Therefore, this observation was also in an agreement with previous studies concerning the stability of reduced aminothiols and HTL in human biofluids ex vivo, as the analytes tend to be more stable in acidic solutions rather than alkaline [16,27,28,33,34].

#### 2.1.4. Derivatization

**Figure 4:**
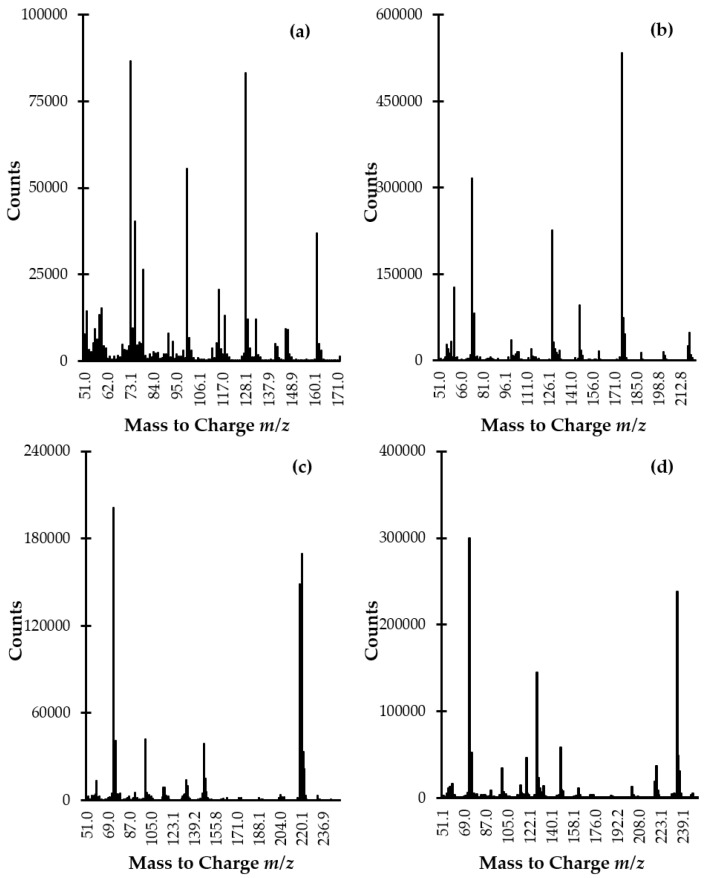
EI scan mode mass spectra of (**a**) HTL-, (**b**) Met-, (**c**) Cys-, and (**d**) Hcy-TMS derivatives obtained by analyzing standard solution of Met, Hcy, HTL, and Cys (100 µmol L^−1^) prepared according to the procedure described in Section 3.5.

It has long been known that the studied analytes are highly polar compounds that are not sufficiently volatile in the original form to meet the requirements of the GC-MS technique. So far, a few GC-MS methods have been described for the determination of Met-related sulfur-containing compounds in plasma and urine [6,7,8,9,10,11,12,13,14,15]. In all cases, a rather simple and efficient way to overcome the above-mentioned constraints, based on modification of the compounds under study into derivatives, has been employed. In the present study, the use of silylation reagent MSTFA purchased as a solution containing 1% TMCS was evaluated. In relation to Met, HTL, and aminothiols, the silylation involves the simultaneous replacement of the active hydrogen on carboxyl (-COOH), -SH, and amino (-NH_2_) groups with trimethylsilyl (-Si(CH_3_)_3_) group resulting in less polar, more volatile and thermally more stable derivatives. Importantly, we found that chemical modification was also valuable from the standpoint of increasing the potential of electron ionization (EI) MS as gave rise to desired derivatives producing intense specific fragment-ion peaks, suitable for the analytes monitoring (Figure 4a–d). With respect to the reduced form of aminothiols, the derivatization was also essential to prevent oxidation leading to alterations in their content.

Additional experiments were performed in order to optimize derivatization conditions. In each case, the appearance of a particular product peak on chromatogram and a comparison of its height was used to determine the reaction’s progress and efficiency, as well. According to the manufacturer’s instruction [35], silyl reagents are influenced by both the solvent system and the addition of a catalyst, which may be required for dissolution of the sample and/or increasing the reactivity of the reagent itself. Most commonly, pyridine is used as a solvent as it is both non-protic and catalyst facilitating the reaction. In some cases, the derivatization reagent can serve as the solvent, as well. Since pyridine is considered to be toxic, preliminary experiments were conducted to check whether its presence is essential for the derivatization reaction. Two sets of samples were prepared where the residue was treated with a mixture containing silylation MSTFA-TMCS reagent and pyridine or derivatizing reagent itself. It has been recognized that TMS derivatives were formed in both cases indicating that the reaction with MSTFA-TMCS mixture does not require any solvents. Nevertheless, more satisfactory results were obtained when the mixture consisting of MSTFA, TMCS, and pyridine was simultaneously subjected to samples. It was found that the signals were higher by about 35–45% under discussed conditions. For this reason, MSTFA-TMCS pyridinic solution was chosen. Such an approach was also advantageous as provided good repeatability of the reaction. Based on our previous studies concerning GC-MS assay dedicated to salivary and urinary HTL measurements [16], freshly prepared derivatizing mixtures were also used, where the ratio of pyridine to MSTFA with 1% TMCS was 1:1.

**Figure 5:**
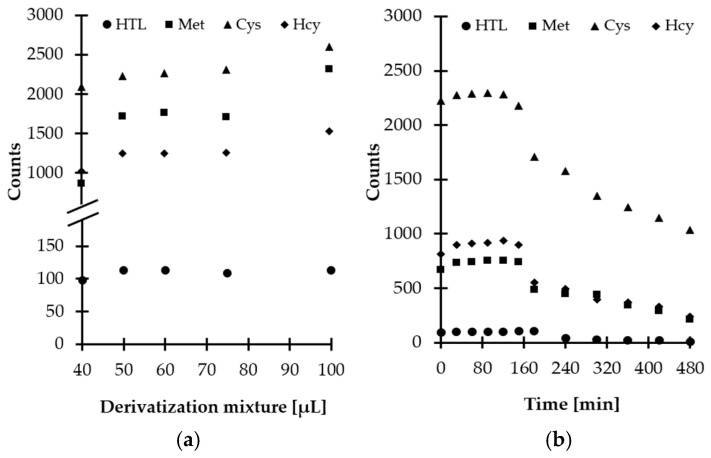
Derivatization reaction yield as a function of (**a**) reagent excess, (**b**) time combined with examination of HTL/Met/Cys/Hcy-TMS derivatives stability in autosampler, expressed as a peak height of HTL/Met/Cys/Hcy-TMS derivatives. Samples were analyzed according to the procedure described in Section 3.6.

Then, experiments were performed to establish the optimal amount of derivatizing mixture for Met-related sulfur-containing compounds derivatization in saliva. Five different quantities, namely, 40, 50, 60, 75, and 100 µL, were tested. Based on our previous studies [16], a smaller amount of derivatizing mixture was not taken under consideration, while a higher volume of MSTFA-TMCS pyridinic solution was excluded in order to prevent further analyte dilution and eliminate the source of baseline noises. As a result, it was found that the best results were obtained when 60 µL of the derivatizing mixture was taken (Figure 5a).

Additional experiments were conducted to establish the derivatization reaction kinetics at room temperature. Importantly, it was recognized that reaction time did not vary greatly among compounds and the reactions at the above-defined conditions were completed in a matter of minutes after mixing of the reagents (Figure 5b). Therefore, no experimental work was undertaken to further optimize the temperature of the reaction. Finally, 5 min reaction time was chosen, as this was considered well within the timeframe required for processing of the samples.

Since chemical compounds can be decomposed prior to chromatographic analysis under different circumstances, the stability of obtained derivatives was also evaluated. Notably, it was found that HTL-TMS and Met/Cys/Hcy-TMS derivatives remain stable up to 180 and 150 min, respectively, at room temperature and then are gradually decomposed owing to hydrolytic instability (Figure 5b). Similar behavior was observed when salivary and urinary HTL was studied [16]. Hence, in order to produce meaningful results, this fact cannot be neglected in any attempt to measure Met-related sulfur-containing compounds content using the proposed GC-MS assay. Moreover, it is highly recommended to analyze samples without delay.

**Figure 6:**
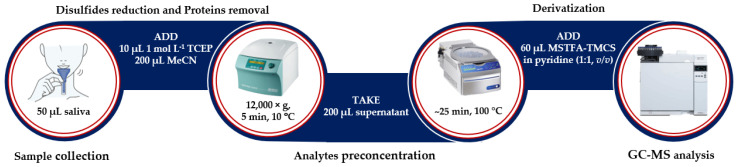
The experimental procedure for HTL, Met, Cys, and Hcy simultaneous determination in human saliva (see Section 3.5).

Finally, our experiments have established an optimal procedure, in which reduced and deproteinized saliva is evaporated to dryness, treated with a freshly prepared derivatizing mixture of MSTFA and TMCS in anhydrous pyridine, and subjected to the GC system (Figure 6). The overall sample preparation time was estimated to be 45 min. In general, the duration of sample pretreatment procedure was similar to results previously reported by other research groups [28].

### 2.2. GC Separation and MS Detection

In the presented study, the initial experiments were conducted with the use of GC-MS method dedicated to salivary and urinary HTL measurements [16]. It has been recognized that most analytes were not well-resolved under isothermal temperature conditions, thus a temperature program was used. Moreover, multiple ramp rates were applied to affect smaller regions of the chromatogram, providing a better resolution of the peaks eluting in the middle of the chromatogram and later eluting peaks, as well. During method development, crucial rules have been pointed out to be followed in order to perform a successful analysis. Importantly, it has been recognized that a starting temperature should be no higher than 146 °C, and maintaining isothermal temperature conditions for 5 min was essential to provide resolution of the HTL-TMS derivative from other saliva constituents. Moreover, it was found that a ramp to 300 °C followed by slow cooling down was necessary to protect the column against contamination and to equilibrate the GC-MS system between analyses. This standard procedure was also essential to eliminate carryover between samples. Under optimized conditions (see Section 3.6), the peaks of TMS derivatives eluted within 10 min and were well-separated, from the responses of all concomitant matrix components, on the capillary column coated with HP-5MS phase.

The identification and confirmation of the target compound were performed by analyzing the standard solution of the analytes (100 µmol L^−1^) prepared according to the procedure described in Section 3.5. Each solution of sulfur-containing compound was prepared separately and then was processed. The GC-MS spectra were initially acquired in scan mode and subsequently in selected ion monitoring (SIM) mode. In the beginning, the instrument was set to gather data stepping the mass filter within *m*/*z* 50–1000 range. The NIST Mass Spectral Library and EI scan mass spectra were used to identify compounds and spectrum peaks. Two ions per target compound were identified as suitable for analyte monitoring (Figure 4a–d, Table 1). Then, analyses were conducted with SIM MS mode in order to increase sensitivity and selectivity in trace analysis. Moreover, signals to be monitored were grouped into time programmed SIM groups to enhance the accuracy and precision of quantitative results. Identification and quantification of the compound of interest in real samples were based upon a comparison of retention time and specific ions with a corresponding set of data obtained by analyzing authentic compounds. The order of elution was as follows: HTL, Met, Cys, and Hcy being in agreement with increasing, theoretically calculated molecular masses of their TMS derivatives. This phenomenon was indirect evidence that all present in the corresponding molecule active hydrogens on -COOH, -SH, and -NH_2_ group were substituted by TMS group since, in relation to GC technique, it is widely known that compounds generally elute in the increasing order of their molecular masses.

**Figure 7:**
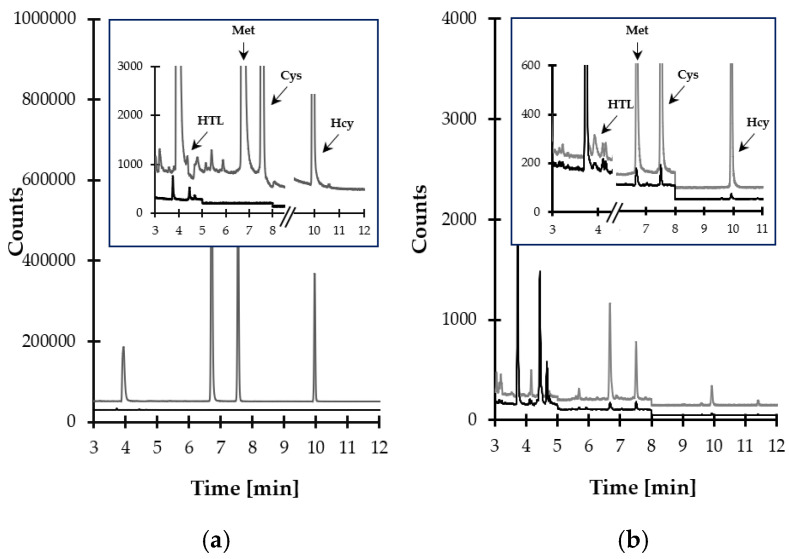
Representative chromatograms of standard solutions and human saliva prepared according to the procedure described in Section 3.5. Chromatographic conditions were as described in Section 3.6. (**a**) Blank standard solution (black line) and standard solution of HTL, Met, Cys, and Hcy (10 µmol L^−1^) (grey line); (**b**) normal human saliva sample (black line) and the same sample spiked with the analytes (10 µmol L^−1^) (grey line).

Finally, satisfactory method selectivity was achieved through the selection of temperature program and the specific ions to be monitored by MS detector. The representative SIM-chromatograms are shown in Figure 7a,b. The data on the retention time of particular target compounds and ions used for their identification and quantification are presented in Table 1.

### 2.3. Validation of the Method

The GC-MS assay was thoroughly validated on a qualified instrument in order to establish that the performance characteristic of the procedure meets the requirements for the intended analytical application. The elements and acceptance criteria of the method development and validation were selected upon the United States Food and Drug Administration guidance for bioanalytical methods validation [36]. The validation protocol included fundamental parameters such as selectivity, linearity, the limit of quantification (LOQ), accuracy, and precision. In general, these parameters were measured in combined experiments. Moreover, system suitability parameters such as repeatability of retention time expressed as the coefficient of variation (CV) of retention time, asymmetry factor, and number of theoretical plates were selected. System suitability test calculations were performed by analyzing a set of standard solutions of HTL, Met, Cys, and Hcy (10 µmol L^−1^) in 10 replicate injections. Importantly, successful system suitability test runs indicated the proper performance of the instruments. The data regarding the system suitability tests are summarized in Table 2.

In the beginning, some attempts have been made to verify the selectivity of the analytes in the presence of concomitant matrix components. In particular, selectivity studies assessed interferences originating from structurally and physiologically similar thiol compounds. These included biologically relevant Met-related aminothiols, such as glutathione (GSH) and cysteinyl-glycine (CysGly), which have been recognized to be also present in saliva specimen so far [28]. At first, blank standard solution and standard solution of HTL, Met, Cys, and Hcy (10 µmol L^−1^) were analyzed. Moreover, each target compound solution was prepared separately in order to ascertain that a single analyte did not yield more than one chromatographic peak. As shown in Figure 7a, the elution profile is free from any interferences at the retention time of the analytes. Importantly, the same observations were made when the standard solution of GSH and CysGly (10 µmol L^−1^) were assayed. Then, blank saliva samples from six individual sources and the same samples spiked with GSH and CysGly (10 µmol L^−1^) were analyzed according to the procedure described in Section 3.5 and Section 3.6. No increase in peak height of the target compounds was observed. In addition, selectivity studies encompassed the evaluation of peak purity. For this purpose, the MS detector was set to acquire spectra on-line throughout the entire chromatogram, and the spectra registered during the elution of each target compound peak were compared. Importantly, the same spectra, acquired in different sections of a particular analyte peak, were observed, indicating its purity.

A standard approach was applied for the calibration of the method. Multilevel calibration curves were generated for each analyte and were run in triplicate over five subsequent working days. The calibration curves consisted of a blank sample and seven calibration standards which concentrations were chosen on the basis of the concentration range expected in study samples [16,24,28]. Calibrators were prepared in laboratory-made pooled saliva by spiking the matrix with known quantities of the analytes. Since saliva samples free of HTL, Met, Cys, and Hcy were not available, the endogenous concentrations of the analytes were evaluated before calibration curve preparation by triplicate analysis. The linearity was initially evaluated graphically by visually inspecting a plot of the peak height as a function of the analyte concentration. Then, the mathematical evaluation was conducted using the least-squares regression model to describe the concentration–response relationship. In particular, curves’ correlation coefficient (R) was monitored showing that the instrument response was directly proportional to the analytes’ concentration within the intended quantitation range. Moreover, substantial changes in the slope of the particular regression line across a day were not observed. Nevertheless, it has been encountered that the analytical method might have been affected by matrix components. Thus, matrix effects were investigated during the validation and implementation of the method. The matrix effect evaluation involved comparing calibration curves in multiple sources of the saliva samples against a calibration curve in the pooled matrix. Importantly, it was recognized that calibration curves created from a pooled matrix did not differ substantially from the ones prepared in human saliva samples from six individual sources. In particular, the slope of the regression lines did not deviate by more than 12.9%. In fact, this denoted the absence of any matrix effect and indicated that most of the interfering matrix components were eliminated among optimized sample preparation procedures. Indeed, with this difference in slope, there only would be a few percent errors in using any of the regression lines to quantify the sample. On the other hand, when dealing with trace amounts, these errors can have a large effect on the analytical results. Due to this variation, a standard addition method was, nonetheless, used.

Accuracy and precision of the assay were evaluated under described conditions in order to assess variability associated with measurements. The precision was expressed in the form of CV, whereas accuracy as the percentage of analyte recovery calculated by expressing the mean measured amount as a percentage of added amount using the following formula:

Accuracy (%) = [(measured amount − endogenous content)/added amount] × 100(1)

The evaluation of the above-mentioned parameters was carried out at two levels, and experiments were completed as a part of linearity assessment. Intra-assay precision and accuracy were demonstrated by triplicate analysis of freshly prepared calibrators. They referred to polled saliva samples spiked with the analytes at three different levels, covering quantitation range, including one close to the LOQ, one in the middle of the range, and one at upper LOQ. Experiments for estimating intermediate accuracy and precision were repeated, in the same manner, over five subsequent days. All concentrations were tested with the use of calibration curves prepared especially on that occasion. Importantly, obtained results from analytical runs met the acceptance criteria. The accuracy ranged from 88.75% to 112.92% and 91.37% to 107.58% for intra- and inter-day variation, respectively. The precision did not exceed 14.30% of CV at any examined concentration level. It varied from 3.60% to 14.30% and 4.87% to 13.72% for intra- and inter-day measurements, respectively. Detailed data on precision and accuracy from the five-day experiments, compared with intra-assay precision and accuracy, are gathered in Table 3.

The LOQ was determined experimentally by the signal-to-noise method. For this purpose, a proxy matrix (0.9% NaCl in 0.1 mol L^−1^ phosphate buffer, pH 7.4) was enriched with decreasing concentrations of the analytes and treated according to the procedure described in Section 3.5 and Section 3.6. In the present study, LOQ was taken as the concentration that resulted in a peak 10 times as high as the baseline noise level, which was clearly distinguished from the baseline and reproductible. The estimated LOQ equals to 0.1 µmol L^−1^ for Met, Hcy, Cys while its value for HTL amounts to 0.05 µmol L^−1^. These concentrations of the analytes produced a detector response with a precision that did not exceed 13.72%, and accuracy ranged from 89.46% to 103.67%. The obtained LOQ values were similar to those published earlier concerning the determination of HTL as well as total Hcy and Cys content in human saliva [16,28].

Finally, the method validation proved that the optimized GC-MS assay is suited to the analysis of the study samples. In particular, it has been demonstrated that the analytical procedure is sensitive enough and has suitable levels of precision and accuracy, falling within acceptable tolerance limits. Detailed data dealing with all validation parameters are shown in Table 3 and Table 4.

### 2.4. Application of the Method

In order to establish the utility of the method, saliva samples from ten apparently healthy volunteers (7 women and 3 men, 24 to 62-year old, providing an average age for the experimental group of 33.70) were analyzed using the GC-MS assay. The average age was 43.00 for men and 29.71 for women. Samples were handled according to the procedures described in Section 3.5 and Section 3.6. A single standard addition method was used to establish salivary levels of Met-related sulfur-containing compounds. Study samples with concentrations above the upper LOQ were diluted and re-analyzed. Concentrations of salivary Met, Cys, and Hcy varied from 12.07 to 51.0 µmol L^−1^ (23.98 ± 16.22 µmol L^−1^), from 3.73 to 16.63 µmol L^−1^ (7.67 ± 4.32 µmol L^−1^), and from 0.32 to 1.67 µmol L^−1^ (0.99 ± 0.54 µmol L^−1^), respectively. HTL was not detected in study samples, most probably due to the limited stability of HTL in saliva ex vivo [16]. However, it needs to be clearly emphasized that in the present study performance of the method was verified using samples collected in 2016. At first glance, these values were remarkably different from thiol levels previously reported using CMLT-based methodology [24]. In relation to samples tested in 2016, concentrations of salivary Cys and Hcy varied from 1.68 to 12.56 µmol L^−1^ (6.01 ± 3.79 µmol L^−1^), and from 0.26 to 1.25 µmol L^−1^ (0.76 ± 0.42 µmol L^−1^), respectively. Therefore, additional experiments were conducted to establish if the GC-MS assay meets performance requirements. The same saliva samples from donors were assayed by methods developed in our earlier studies [16,24]. Aminothiols, namely Hcy and Cys were quantified by a method using HPLC with pre-column derivatization with CMLT and UV detection [24]. The assay based on the GC-MS technique, designated for assessment of HTL content in human saliva and urine, was used for its determination [16]. To the best of our knowledge, no reference method was available to estimate Met content in saliva specimen and the presented GC-MS assay is the first one dealing with the above-mentioned issue. Importantly, the values for saliva Met-related sulfur-containing compounds were similar to those obtained using a newly developed GC-MS assay. In relation to Cys and Hcy, the results did not deviate by more than 11.9%, indicating the procedure’s reliability. The greatest possible reason for the difference in levels of analytes, if compared to 2016 results, was some sample components decomposition or partial evaporation as evaluated values were about 30% higher than those estimated in 2016. On the other hand, these studies have confirmed the supposition that the stability of the analytes in saliva ex vivo is limited. According to literature data [28], there are no reliable experimental data on the long-term stability of aminothiols and HTL in saliva, as yet. Thus, this topic remains to be investigated in detail in the near future to ensure that samples are not exposed to decomposition upon handling process.

## 3. Materials and Methods

### 3.1. Reagents and Materials

All chemicals used throughout this study were of analytical reagent grade. D,L-HTL, D,L-Met, CysGly, symmetrical disulfides of D,L-Hcy, D,L-Cys, and L-GSH, MSTFA, TMCS, TCEP, DTT, 2-ME, THP, HSA, sodium chloride, and anhydrous pyridine were from Sigma-Aldrich, (St. Louis, MO, USA). PCA, hydrochloric acid, acetic acid, sodium hydroxide, HPLC-gradient grade MeCN, ethanol, chloroform, methanol, sodium hydrogen phosphate heptahydrate, sodium dihydrogen phosphate dihydrate were from J.T. (Baker, Deventer), the Netherlands. CMLT was prepared as previously described [24]. Deionized water was produced in our laboratory.

### 3.2. Instrumentation

An Agilent 7820A GC system equipped with automated sample injector model 7693A and MS detector 5977B (Agilent Technologies, Waldbronn, Germany) was used for GC experiments. The GC apparatus was equipped with a split/splitless inlet, working in a split ratio of 50:1 mode to a 30 m × 0.25 mm HP-5MS quartz capillary column with a 0.25 µm film thickness (Agilent Technologies, Waldbronn, Germany). Data acquisition and analysis were performed using MassHunter 5977B MSD Bundle with 7820 GC software NIST MS Spectral Library version 2.3.

HPLC analyses were carried out using an Agilent 1220 Infinity LC system equipped with a binary pump integrated with two-channel degasser, autosampler, column oven, and diode-array detector (Agilent Technologies, Waldbronn, Germany) controlled by OpenLAB CDS ChemStation software. Analytes were separated on Aeris PEPTIDE XB-C18 (150 mm × 4.6 mm, 3.6 µm) column from Phenomenex, Torrance, CA, USA.

For absorbance measurements, the UV-1900 spectrophotometer with the UVProbe software (Shimadzu, Kyoto, Japan) was used. Samples were dried using a CentriVap Centrifugal Vacuum Concentrator (Labconco, Kansas City, MO, USA). For sample shaking, Multi-Speed Vortex MSV-3500 (Biosan, Riga, Latvia) was used. During the study, a Mikro 220R centrifuge with fast cool function (Hettich Zentrifugen, Tuttlingen, Germany), an HI 221 pH-meter (Hanna Instrument, Loveland, CO, USA), and a QBD2 thermostat (Grant Instruments Ltd., Cambridge, UK) were also exploited. Samples were stored in an ultra-low-temperature freezer (Panasonic Healthcare Co., Ltd., Sakata, Japan). Water was purified using a Milli-QRG system (Millipore, Vienna, Austria).

### 3.3. Stock Solutions

The stock solution of TCEP (1 mol L^−1^) was prepared by dissolving an appropriate amount of TCEP powder in deionized water. The solution of TCEP was prepared freshly and was processed without delay. Stock solution of HSA (5 g L^−1^) was prepared in 0.9% NaCl as needed. Stock solutions of 0.1 mol L^−1^ Met, HTL, and symmetrical disulfides of Hcy and Cys were prepared in 1 mol L^−1^ HCl. These solutions were kept at 4 °C for no longer than 7 days without noticeable change of the analyte content. The working solutions of the analytes were prepared daily by dilution of a standard solution with deionized water.

### 3.4. Biological Samples Collection

First, early morning saliva samples (about 2 mL) were collected from individuals after overnight fasting and before teeth brushing. Unstimulated saliva samples were obtained by asking donors to put whole expectorated oral fluid into sterile tubes. Then, samples were cooled on ice and delivered to the laboratory, where samples were stored at −80 °C until analysis. Samples were processed immediately after defrosting at room temperature using the procedure described in Section 3.5.

In the present study saliva samples, stored at −80 °C since 2016, were tested. Such an approach was due largely to the SARS-CoV-2 pandemic. These samples were collected for the purpose of demonstrating the validity of CMLT-based methodology [24]. Ten apparently healthy volunteers, belonging to an ethnically homogeneous group, were studied. Donors were not supplemented with analytes before sample collection. No medications were also allowed. The study was approved by the Ethical Committee of the University of Lodz (decision identification code: 2/KBBN-UŁ/III/2020, date 16 April 2020). All subjects gave their informed consent.

### 3.5. Saliva Specimen Preparation for Met-Related Sulfur-Containing Compounds Quantification by GC-MS

In the beginning, saliva was clarified by centrifugation (12,000× *g*, 10 min, 4 °C). Then, 50 µL of obtained supernatant was mixed with 200 µL of MeCN and 10 µL of 1 mol L^−1^ TCEP. The mixture was vigorously shaken and kept in a centrifuge at 12,000× *g* for 5 min at 10 °C. After centrifugation, the upper organic layer (200 µL) was transferred into a 0.5 mL polypropylene microtube and dried under vacuum (~25 min at 100 °C). Thereafter, the residue was treated with 60 µL derivatizing mixture containing MSTFA with 1% TMCS in pyridine (1:1, *v*/*v*) and incubated at room temperature for 5 min. Afterward, the reaction mixture was transferred to a vial, and 1 µL of the sample was injected into GC-MS system without delay. Each sample was analyzed according to the procedure described in Section 3.6.

### 3.6. GC-MS Conditions

Helium (99.9999%) was used as the carrier gas with a constant flow rate of 1 mL min^−1^. The injection port temperature was set to 280 °C. The chromatographic separation of Met-related sulfur-containing compounds, TMS derivatives, was accomplished under thermal gradient conditions. The initial oven temperature was set to 146 °C for 5 min and increased to 200 °C in steps of 5 °C min^−1^, then 50 °C min^−1^ to 300 °C. Then, the oven was slowly cooled down in steps of 20 °C min^−1^ afterward. The MS detector was operated in the EI mode at 70 eV. The ion source temperature was set at 230 °C, the temperature of quadrupole was set at 150 °C, while the MS interface was set to 250 °C. The multiple ion detector was focused on ions that represent only the portion of each target compound. These ions were grouped into three time-programmed SIM groups and the instrument was set to acquire data with a dwell time that yields 15 to 20 scans across the chromatographic peak. Information concerning the SIM MS mode of detection is gathered in Table 1.

## 4. Conclusions

To the best of our knowledge, this is the first and the only available GC-MS assay dedicated to salivary Met-related sulfur-containing compounds measurements. The method allows simultaneous determination of a number of salivary biologically relevant compounds, namely, Met, total Cys, and Hcy as well as HTL in a single run. In addition, this is the first-ever report dealing with the presence of Met in the above-mentioned matrix as well as the first effective analytical tool enabling to determine Met in human saliva. The assay is primarily characterized by a streamlined sample preparation procedure involving simultaneous disulfides reduction with deproteinization, analytes preconcentration by drying under vacuum, and derivatization followed by GC-MS analysis. Moreover, relatively short analysis time and the possibility of carrying out chemical analysis on a very small scale combined with low consumption of hazardous chemicals and laboratory disposable plasticware make the GC-MS assay environmentally friendly. Unfortunately, the method is not free from restrictions. In order to produce meaningful results, it is highly recommended to quantify the samples using the standard addition method, which limits the number of samples analyzed per day and assay them without delay due to the limited stability of TMS derivatives under experimental conditions. Nevertheless, we hope that the proposed GC-MS assay will (1) facilitate investigations into the clarification of the role of HTL, Met, Cys, and Hcy in physiological and pathological states in living systems, as well as (2) contribute substantially to broaden our knowledge of the diagnostic potential of saliva.

## Figures and Tables

**Table 1:** Analytical characteristics of HTL/Met/Cys/Hcy-TMS derivatives using SIM MS mode.

Analyte		HTL	Met	Cys	Hcy
Retention time	(min)	3.9	6.7	7.5	9.9
CV of retention time (*n* = 10)	(%)	0.442	0.060	0.049	0.038
Identification ions	(*m*/*z*)	128.1; 161.1	128.1; 176.1	218.1; 220.1	128.1; 234.1
Quantification ions	(*m*/*z*)	128.1	176.1	220.1	234.1
Time programmed SIM group		1st	2nd	2nd	3rd
SIM group start time	(min)	2.2	5.0	5.0	8.0

**Table 2:** System suitability test (*n* = 10).

Analyte		HTL	Met	Cys	Hcy
**Acceptance Criteria**		**Value**			
CV of retention time	≤1%	0.442%	0.060%	0.049%	0.038%
Assymetry factor	0.8–1.5	1.39	1.47	1.16	1.50
Number of theoretical plates	≥2000	10,384	11,375	22,278	19,274

**Table 3:** Precision and accuracy data (*n* = 5).

Analyte	Concentration (µmol L^−1^)	Precision (%)		Accuracy (%)	
		Intra-Assay	Intermediate	Intra-Assay	Intermediate
HTL	1	12.80	11.77	89.46	92.53
	10	8.53	12.24	102.21	96.91
	20	9.46	10.98	96.04	101.51
Met	1	14.30	13.72	91.54	102.74
	10	5.75	7.83	88.75	107.58
	20	10.69	9.54	100.41	101.96
Cys	1	9.87	11.23	112.92	91.37
	10	3.60	5.89	103.15	104.15
	20	7.83	6.24	105.09	99.87
Hcy	1	10.60	12.36	99.59	103.67
	10	6.58	4.87	96.79	94.53
	20	12.03	9.56	102.91	100.22

**Table 4:** Validation data (*n* = 3).

Analyte	Regression Equation	R (R^2^)	CV Slope (%)	Linear Range (µmol L^−1^)	Intra-Assay Precision (%)		Intra-Assay Accuracy (%)		LOQ (µmol L^−1^)
					Min	Max	Min	Max	
HTL	y = 2.935x + 1.890	0.9970 (0.9940)	1.9	1.0–20	8.53	12.80	89.46	105.17	0.05
Met	y = 26.10x + 43.51	0.9940 (0.9880)	3.5	0.5–20	5.75	14.30	88.75	106.35	0.1
Cys	y = 70.31x + 5.908	0.9952 (0.9904)	1.4	0.5–20	3.60	11.57	94.93	112.92	0.1
Hcy	y = 28.94x + 26.63	0.9969 (0.9938)	1.2	0.5–20	1.52	13.54	94.63	102.91	0.1

## Abbreviations

CMLT	2-chloro-1-methyllepidinium tetrafluoroborate
Cys	cysteine
CysGly	cysteinyl-glycine
CV	coefficient of variation
DTT	dithiothreitol
EI	electron ionization
GC	gas chromatography
GSH	glutathione
Hcy	homocysteine
HPLC	high-performance liquid chromatography
HSA	human serum albumin
HTL	homocysteine thiolactone
LOQ	limit of quantification
MeCN	acetonitrile
Met	methionine
MS	mass spectrometry
MSTFA	N-trimethylsilyl-N-methyl trifluoroacetamide
PCA	perchloric acid
R	correlation coefficient
SD	standard deviation
SIM	selected ion monitoring mode
TCEP	tris(2-carboxyethyl)phosphine
THP	tris(hydroxymethyl)phosphine
TMCS	trimethylchlorosilane
TMS	trimethylsilyl group
UV	spectrophotometric detection
2-ME	2-mercaptoethanol
